# Analysis of Historic Copper Patinas. Influence of Inclusions on Patina Uniformity

**DOI:** 10.3390/ma10030298

**Published:** 2017-03-16

**Authors:** Tingru Chang, Inger Odnevall Wallinder, Daniel de la Fuente, Belen Chico, Manuel Morcillo, Jean-Marie Welter, Christofer Leygraf

**Affiliations:** 1 KTH Royal Institute of Technology, Div. Surface and Corrosion Science, School of Chemical Science and Engineering, SE 10044 Stockholm, Sweden; tingru@kth.se (T.C.); ingero@kth.se (I.O.W.); 2 National Centre for Metallurgical Research (CENIM-CSIC), 28040 Madrid, Spain; delafuente@cenim.csic.es (D.d.l.F.); bchico@cenim.csic.es (B.C.); morcillo@cenim.csic.es (M.M.); 3 Independent scholar, Luxembourg-1361, Luxembourg; jean-marie.welter@pt.lu

**Keywords:** atmospheric corrosion, historic copper, patina, bilayer, cuprite, brochantite, antlerite, inclusions, Volta potential, micro-galvanic effect

## Abstract

The morphology and elemental composition of cross sections of eight historic copper materials have been explored. The materials were taken from copper roofs installed in different middle and northern European environments from the 16th to the 19th century. All copper substrates contain inclusions of varying size, number and composition, reflecting different copper ores and production methods. The largest inclusions have a size of up to 40 μm, with most inclusions in the size ranging between 2 and 10 μm. The most common element in the inclusions is O, followed by Pb, Sb and As. Minor elements include Ni, Sn and Fe. All historic patinas exhibit quite fragmentized bilayer structures, with a thin inner layer of cuprite (Cu_2_O) and a thicker outer one consisting mainly of brochantite (Cu_4_SO_4_(OH)_6_). The extent of patina fragmentation seems to depend on the size of the inclusions, rather than on their number and elemental composition. The larger inclusions are electrochemically nobler than the surrounding copper matrix. This creates micro-galvanic effects resulting both in a profound influence on the homogeneity and morphology of historic copper patinas and in a significantly increased ratio of the thicknesses of the brochantite and cuprite layers. The results suggest that copper patinas formed during different centuries exhibit variations in uniformity and corrosion protection ability.

## 1. Introduction

The production and use of copper and copper alloys is many thousands of years old and has been based on several different production technologies over the millennia [1]. In early times, native copper and copper extracted from oxide ores was obtained through a reduction with charcoal. These ores were rapidly exhausted in Europe, and ancient metallurgists had to learn to obtain the copper from a large variety of copper sulfide ores. They range from rather pure chalcopyrite (CuFeS_2_) to the *Fahlerz* ore with high antimony and arsenic contents. Well-known *Fahlerz* deposits were exploited until the 19th century in Schwaz (Austrian Tirol) and Neusohl (Slovakia), which belonged to the Austro-Hungarian Empire. Often the copper ore was a minority component in an ore body. In the mine of Falun (Sweden), for instance, lead and zinc sulfides were the main constituents. After this, oxygen became the agent that helped to reduce sulfur and other major and minor elements contained in the ore. This explains why old copper samples contain larger amounts of oxygen (ranging from 400 to 4000 ppm by weight) and other metallic impurities. The most important one is lead, with contents of up to 1% by weight, followed by arsenic and antimony, often up to 0.5% by weight. Further minor elements included nickel, silver, tin, zinc, iron, and bismuth. It is only at the end of the 19th century that progress in electro-refining resulted in a total impurity content well below 1000 ppm by weight. A few decades later, the emergence of acetylene torches led to an almost complete desoxidation of copper intended to be joined by brazing and welding. Nowadays, phosphor is the preferred desoxidant and some 200 to 300 ppm by weight is left in copper used to fabricate e.g., roofing sheets and water tubes. This brand is named *Desoxidized high phosphor* copper (DHP-Cu). During its annealing—even at reduced oxygen partial pressures—some oxygen diffuses into the bulk and forms with phosphor a thin sub-surface layer of copper meta-phosphate, which will modify the corrosion behavior of the copper [2]. Sheets of copper were manufactured until the end of the 18th century by hammering, after which rolling was made possible by the development of large and strong mills [3]. A great deal of our knowledge of early production methods originates from sophisticated characterizations of copper and copper alloy artifacts [4,5,6]. It also originates from detailed analysis of their inclusions [7,8], which to some extent can reveal the nature of the copper ores from which the copper was extracted. Combined with knowledge about the metallurgy of copper or copper alloys, such studies have contributed to a better understanding of historic metallurgical and foundry processes and also of the ores and, hence, the minerals from which the objects emanate.

This study of selected historic copper materials aims to explore how such objects can reveal information about their history, by analysis not only of the copper substrate, but also of the corrosion product layers that have grown on the surface over extended exposure times. These layers are commonly designated as (natural) patina, although the term also can refer to man-made, chemically induced modifications of the surface appearance. Thus, can an in-depth analysis reveal anything about the environment in which the layers were formed? Chemical characteristics of patina formed on copper alloys as an environmental indicator has been reported before: for instance, the detailed analysis of sulfur isotopes in the sulfur-containing corrosion products suggested that the element could be traced to sulfur contained in air-polluting species, despite large geographic variations in the analyzed samples [9]. An important study to be mentioned in this context is the extensive patina study that was performed as part of the restoration of the Statue of Liberty [10,11]. Two families of inclusions were identified, but until now they have not been related to the structure of the corrosion product layers [12].

The present study includes copper materials that originate from roofs on different historical buildings, eight historic surfaces and one modern commercially available sheet for comparison. The focus of this paper is on the inclusions embedded in the copper matrix. They turn out to vary substantially between the different materials with respect to size, frequency and chemical composition. The underlying question to be explored herein concerns if and how the characteristics of these inclusions may influence the composition and homogeneity of the copper patina formed.

## 2. Materials and Methods

### 2.1. Materials and Sample Preparation

The examined historic copper materials have been exposed for roofing on different historic buildings in variety of environments and exposure times, the oldest at the Royal Summer Palace (Belvedere) in Prague, Czech Republic (completed between 1538 and 1560) and the youngest at a church in the Old Town of Stockholm, Sweden (exposure period around 100 years, starting in the 19th century). Other exposure sites include Drottningholm Castle outside Stockholm, Sweden, the Mausoleum in Graz, Austria, Basilica Maria Dreieichen in Lower Austria, Helsinki Cathedral, Finland, Otto Wagner Church in Vienna, Austria, and Kronborgs Castle, Elsinore, Denmark. Information on exposure site and estimated length of exposure period is given in Table 1.

Cross-sections of the historic copper patinas were prepared by embedding the materials into a conductive polymer followed by polishing using 0.25 μm diamond paste to obtain a near mirror-like cross-sectional surface. Prior to the investigations, all samples were ultrasonically cleaned in analytical-grade ethanol for 10 min and subsequently dried by cold nitrogen gas.

### 2.2. SEM/EDS (Scanning Electron Microscopy and Energy Dispersive Spectroscopy)

SEM/EDS-analysis (scanning electron microscopy and energy dispersive spectroscopy) was conducted to obtain morphology and elemental information. Cross-sections were analyzed using a LEO 1530 instrument (Zeiss, Oberkochen, Germany) with a Gemini column, upgraded to a Zeiss Supra 55 (Zeiss) and an EDS X-Max SDD (Silicon Drift Detector) 50 mm × 50 mm detector from Oxford Instruments (Oxford, UK). All analyses were performed by means of a FEI-XL 30 Series instrument (Oxford, UK), equipped with an EDS system (EDAX Phoenix) with an ultra-thin windows Si-Li detector. All images were obtained using backscattered electrons at an accelerating voltage of 15 kV. The data of EDS elemental composition was collected by Oxford Instruments INCA 5.04.

### 2.3. AFM/SKPFM (Atomic Force Microscopy and Scanning Kelvin Probe Force Microscopy)

AFM-analysis (Nanoscope Icon AFM from Bruker, Karlsruhe, Germany) was employed to image the surface topography and to map Volta potential variations along the cross-section of selected historic copper substrates. The Volta potential mapping was carried out using the Kelvin probe technique [13,14]. For this purpose, a SCM-PIT probe from Bruker and a HQ:NSC18/PT probe from MicroMash (Wetzlar, Germany) were used. The data were processed with Gwyddion (Czech Metrology Institute, Brno, Czech Republic), which is a free modular software program for SPM data visualization and analysis [15].

### 2.4. EIS (Electrochemical Impedance Spectroscopy)

EIS measurements were performed at the open circuit potential (OCP) to estimate the polarization resistance of one historic and one modern, commercially available, copper sheet (DHP-Cu, >99.9 wt % Cu) by using a Multi Autolab (Metrohm Autolab B.V., Utrecht, The Netherlands) instrument. 0.1 M Na_2_SO_4_ was used as the electrolyte with an Ag/AgCl reference electrode and platinum mesh as counter electrode. The applied perturbation amplitude was 10 mV with the measured frequency range of 10^4^–10^−2^ Hz with 75 measuring points. To perform the experiments at oxygen-free conditions, the solution was purged with N_2_ gas for 30 min prior to the experiment. The EIS data was analyzed by using the Nova 1.8 software (Metrohm Autolab B.V., Utrecht, The Netherlands).

## 3. Results

**Figure 1:**
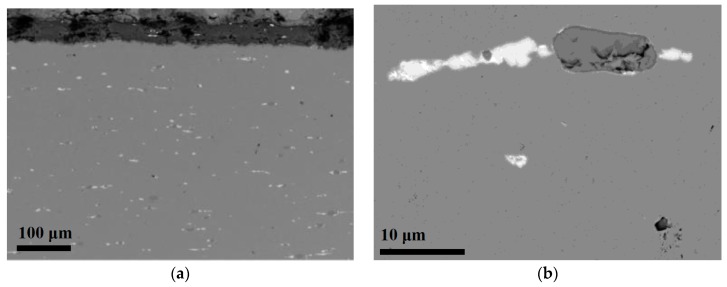
SEM images of an overview cross-section of the historic copper material from the Royal Summer Palace (Belvedere) in Prague showing the copper substrate (lower part in light grey) and the patina (upper part in dark grey) (**a**); Different inclusions in the substrate at higher magnification (**b**).

Figure 1 displays SEM images at lower (a) and higher (b) magnification of the copper sample collected from the Royal Summer Palace in Prague, the oldest of the historic samples investigated. It is evident that the substrate contains numerous inclusions, seen as white spots (lower part in light grey, Figure 1a,b at higher magnification). Most of them exhibit an elongated shape in the hammering or rolling direction of the sheet, which was also observed in historic copper materials from the Middle Ages [16]. The inclusions are also seen within the patina (upper part in dark grey, Figure 1a) and suggest that the inclusions, at least partially, remain intact during the atmospheric corrosion process when the outermost surface of the copper substrate is oxidized and transformed to copper patina. Similar inclusion distributions were previously observed in historical French roofs [17].

**Figure 2:**
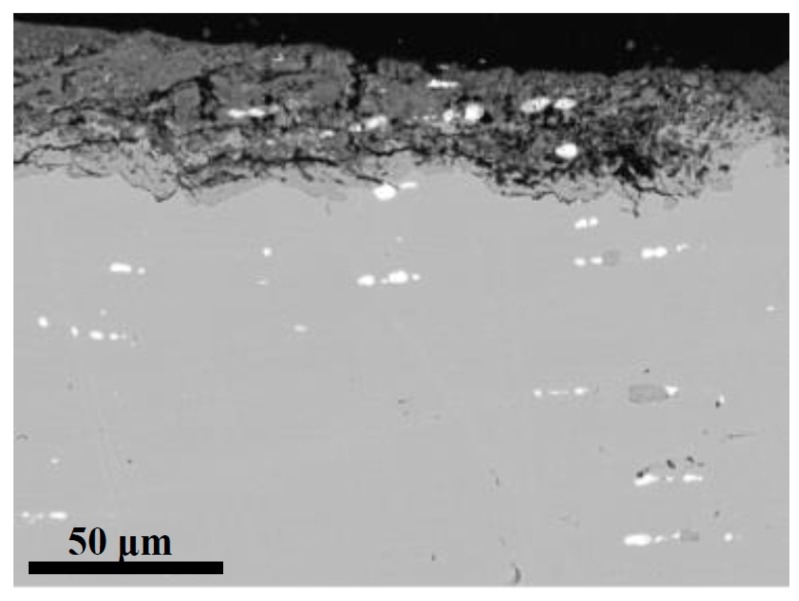
Overview SEM image of a cross-section of the historic copper material from the Drottningholm Castle outside Stockholm showing the copper substrate (lower part, light grey) and the patina (upper part, darker grey). The thin cuprite layer next to the substrate is slightly lighter grey than the outer brochantite layer in darker grey.

Figure 2 is another example of a copper sample (Drottningholm Castle outside Stockholm) with inclusions embedded both in the substrate (lower part in figure, light grey) and in the patina (upper part, darker grey). As in all other samples, the patina consists of two irregularly shaped layers, one closer to the substrate and another, slightly darker, on the outside. It can be seen that the inner layer consists of cuprite (Cu_2_O) and the outer mainly of brochantite (Cu_4_SO_4_(OH)_6_), sometimes partially also of antlerite (Cu_3_SO_4_(OH)_4_).

As seen in Figure 2, the cuprite layer thickness (inner layer) varies considerably along the copper substrate. Moreover, the thickness is generally thinner where the brochantite/antlerite layer contains inclusions (the central part of Figure 2) and thicker where the same layer lacks any evident presence of inclusions. The same tendency is also seen in other historic materials in this study, and suggests that the presence of inclusions may influence the homogeneity of the patina. To explore if the nature of the inclusions can have an impact on the patina formation, an investigation based on SEM/EDS was made of the density, size range and composition of the inclusions observed in all the historic materials. For each copper sample, at least three areas, sized 300 μm × 300 μm, were selected for detailed investigations. The results have been summarized in Table 2.

From Table 2 it is evident that the inclusion characteristics vary considerably between the samples. For the density and size range the intervals shown reflect variations between different investigated areas in the substrate. The density is highest for Drottningholm Castle and Basilica Maria Dreieichen, and lowest for Helsinki Cathedral. The largest inclusion sizes, on the other hand, are seen for the Royal Summer Palace and Helsinki Cathedral, and the smallest for Basilica Maria Dreieichen and Kronborgs Castle.

Regarding the elemental composition, the information in Table 2 is given for a representative selected single larger inclusion rather than for a mixture of inclusions. The most common element in the inclusions is oxygen, suggesting that most inclusions are oxides of different mixtures of metals. The elemental ratio between copper and oxygen in the Kronborgs Castle sample suggests that cuprite (Cu_2_O) is the most probable compound in the inclusion analyzed. Similarly, the results from the Royal Summer Palace sample suggest that the inclusion most likely contains rosiaite (PbSb_2_O_6_), sometimes with smaller cuprite inclusions adjacent to rosiaite inclusions. Separate XRD measurements of inclusions in the patina formed on the copper roof of the Royal Summer Palace confirm that the inclusions, indeed, consist of cuprite and rosiaite [18]. The marked variation in elemental composition of the inclusions analyzed in the different copper samples suggests that several other single or mixed oxides of primarily lead, antimony, copper and tin may be present in the inclusions. A third type of inclusion, different from the others and present in the Graz Mausoleum sample, is a mixture of oxides containing copper, nickel and antimony. Due to their small size, however, it is hard to reveal their exact identity by techniques such as X-ray diffraction, which is able to identify crystalline phases.

The origin of the precipitates is clear. Oxygen is soluble to some extent in liquid copper, but not in the solid phase. When the melt solidifies, oxygen precipitates by forming an oxide. In pure copper—such as modern *Electrolytic tough pitch* copper (ETP copper), containing some 200 to 300 ppm by weight of oxygen—only cuprite is formed. In old copper, the impurities present in large amounts compete with copper to form more or less complex oxides depending on their respective chemical potentials. A special element is lead: like oxygen, it is not soluble in solid copper and precipitates either as a metallic nodule or as a (multi-element) oxide.

**Figure 3:**
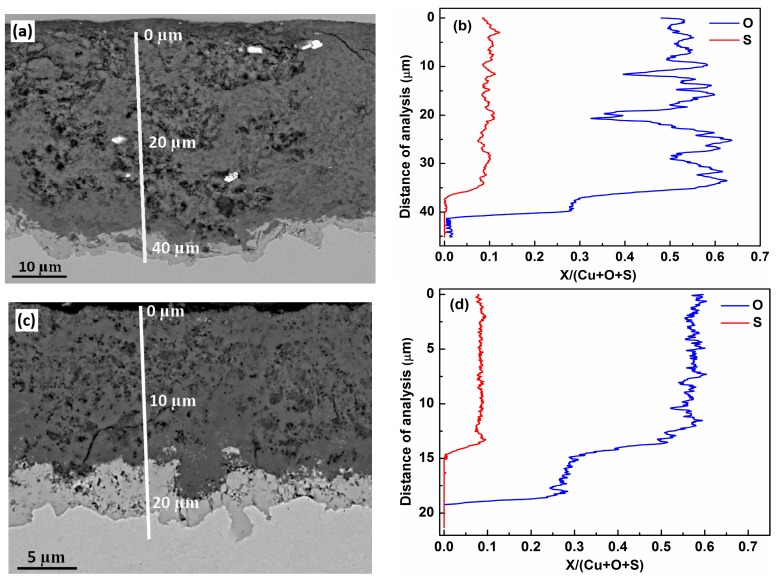
Cross-sections of the patina and adjacent substrate (**a**,**c**) and the corresponding elemental distribution of oxygen and sulfur relative to the sum of (copper+oxygen+sulfur) (in atomic %) based on Energy Dispersive Spectroscopy (EDS) analysis (**b**,**d**) of copper patina formed at the Royal Summer Palace in Prague (**a**,**b**) and at Kronborgs Castle (**c**,**d**), respectively.

We consider next the thickness of the patina formed, in particular the thickness of the cuprite and brochantite/antlerite sub-layers. Figure 3 exhibits the cross-section of the patina and adjacent substrate (a) and the corresponding elemental distribution of oxygen and sulfur relative to the sum of (copper + oxygen + sulfur) (in atomic %) based on EDS analysis (b) of the copper patina formed at the Royal Summer Palace in Prague. It is evident that the relative sulfur- and oxygen-distributions show two levels. An inner sub-layer close to the substrate is characterized by an insignificant sulfur-level and a lower oxygen-level, while an outer sub-layer is characterized by both higher sulfur and oxygen levels relative to the inner sub-layer. There is substantial evidence that this inner layer consists of cuprite and the outer layer of brochantite, antlerite or both, where their formation rate strongly depends on prevailing environmental and pollutant conditions [19,20]. Detailed studies of the evolution of copper patina in different exposure sites have provided great evidence that the inner cuprite layer forms instantaneously when copper is exposed to air, and then continues to grow during extended exposure time. In more sulfur-rich atmospheres, the growth of the patina results in antlerite (Cu_3_SO_4_(OH)_4_) as the end product, with strandbergite (Cu_2.5_SO_4_(OH)_3_·2H_2_O) as a precursor. In less sulfur-rich atmospheres the end product of patina growth is brochantite (Cu_4_SO_4_(OH)_6_), with posnjakite (Cu_4_SO_4_(OH)_6_·H_2_O) as a precursor [20]. In order to distinguish the end products brochantite and antlerite from each other it is necessary to use X-ray diffraction. In the current investigation, which is based on SEM/EDS alone, we denote the outer layer as brochantite/antlerite with no further distinction between the two sulfur-containing phases. However, a more detailed analysis of the patina formed at the Royal Summer Palace in Prague and based on X-ray diffraction is the subject of a later publication [18].

In an analogous way, the results in Figure 3c,d provide evidence of the existence of two sub-layers, cuprite and brochantite/antlerite, for the patina formed at Kronborgs Castle. An interesting difference is that the cuprite layer thickness on average is higher on Kronborgs Castle material than on the material from the Royal Summer Palace.

Table 3 compiles thicknesses of the cuprite and brochantite/antlerite sub-layers determined for the patinas of all historic materials. The average thickness was obtained by measuring the area of each patina layer of five SEM cross-section images (≥90 μm × 65 μm) of each material. A large variation in thicknesses of both sub-layers can be seen. The average cuprite thickness ranges from around 1 μm (Helsinki Cathedral) to around 10 μm (Basilica Maria Dreieichen), while the average brochantite/antlerite thickness varies from less than 10 μm (Helsinki Cathedral) to around 42 μm (Royal Summer Palace). The thickness ratio ((brochantite/antlerite)/cuprite) between the sub-layers also varies with an order of magnitude from 10.6 (Royal Summer Palace) to 1.1 (Otto Wagner Church).

**Figure 4:**
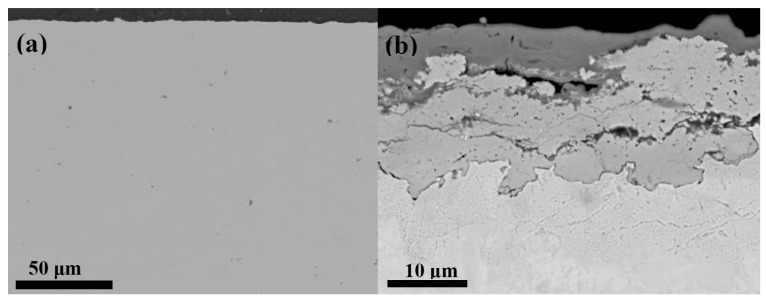
Overview SEM-image of a cross-section of modern sheet copper (*Desoxidized high phosphor* copper (DHP-Cu)) with patina (**a**); More detailed view of cross-section of patina after 5 years in a marine test site of Brest, France (**b**). The inner layer consists of cuprite, the outer layer of atacamite.

It is interesting to compare these ratios with available information from patinas formed on commercial sheet copper during contemporary periods rather than historic ones. Reference [20] contains a rich set of data from sheet copper (DHP-Cu, >99.9 wt % Cu) exposed at 39 exposure sites and exposure times of up to eight years, where the mass of the brochantite, cuprite and other sub-layers have been determined. The brochantite/cuprite thickness ratio from Prague after eight years, for instance, shows a thickness ratio of around 1.25, and corresponding thickness ratios for all other sites also give values of around 1.25 or lower. Therefore the cuprite layer is much more dominating in patinas formed on DHP-Cu than in historic patinas, and they also exhibit more uniform sub-layers than historic patinas. Figure 4a is an example of such modern sheet copper (DHP-Cu, >99.9% Cu, 0.02%–0.1% P, by weight), which is basically free of inclusions (if present < 1 μm) due to its purity. Figure 4b shows the substrate after exposure in a marine test site (Brest, France) for five years. It is evident that the inner cuprite layer has a thickness that clearly exceeds the thickness of the outer layer, in this case atacamite (Cu_2_Cl(OH)_3_). The formation of atacamite is due to the dominance of chlorides in the marine test site of Brest, as opposed to all other sites investigated herein, where the chlorides obviously were not present in sufficiently high concentrations to detect any atacamite.

In all, the results so far show that patinas formed in historic times to a much greater extent are dominated by a brochantite/antlerite layer and by more inhomogeneous and fragmented sub-layers. Patinas formed on commercial DHP-copper, on the other hand, possess a more dominating cuprite layer whereby both sub-layers are characterized by a much higher homogeneity. It should be added that the thickness ratio brochantite/cuprite may change over exposure time for a given exposure situation. This was demonstrated by Fitzgerald et al. for copper exposed in Brisbane, Australia, over a time period of 140 years. The study suggested different growth mechanisms for the two layers: the growth of the inner cuprite layer is controlled by diffusion of cuprous ions through cuprite, while the growth of the outer brochantite layer is controlled mainly by precipitation from the aqueous ad-layer of the ionic species, involved [21].

**Figure 5:**
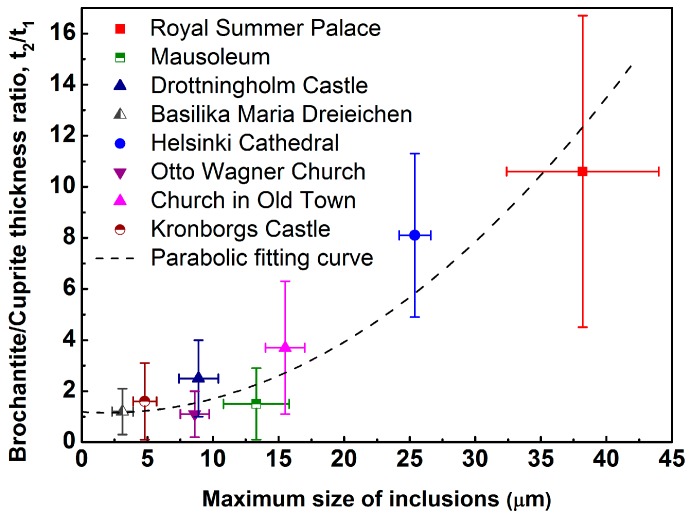
Relation between the thickness ratio of the brochantite/cuprite layer, (t_2_/t_1_), and the observed maximum size of inclusions within the copper substrates of the historic copper materials from the various exposure sites. The dotted parabolic line is only included for guidance–with no physical meaning.

Nevertheless, the question is to what extent the brochantite/cuprite thickness ratio may depend on the copper substrate characteristics, in particular those of the inclusions. By comparing the thickness ratio (right column of Table 3) with data on inclusion properties (Table 2), it can be concluded that neither the inclusion density nor their elemental composition can be used to explain the brochantite/cuprite thickness ratio or the patina homogeneity. However, when plotting the maximum size of the inclusions (Table 2) in the different historic copper materials against the observed brochantite/cuprite thickness ratio, a surprisingly good correlation is obtained (Figure 5).

It is clearly seen that the brochantite/cuprite thickness ratio increases continuously with the maximum size of the inclusions. The error bar for the maximum size is based on the different observed maximum values of inclusion sizes obtained on each investigated 300 μm × 300 μm area, and presented as average values with standard deviation. The figure also shows a dashed line which is the result of a parabolic fit (with no underlying physical meaning) of all data points. When extrapolating the data to a maximum inclusion size of 0 μm, a thickness ratio of around 1.1 is obtained. This value is in good agreement with observations based on patinas formed on sheet DHP-Cu, as mentioned above (see further reference [20]).

## 4. Discussion

**Figure 6:**
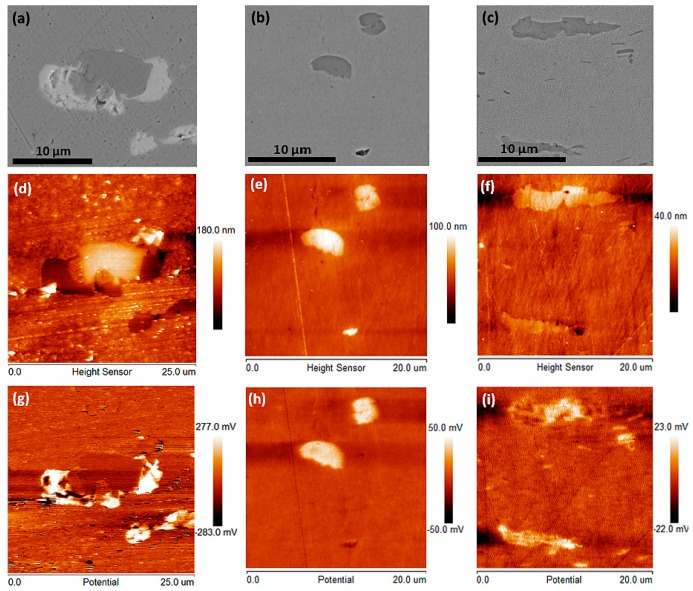
SEM-images (**a**–**c**), the corresponding atomic force microscopy (AFM)-based topographies (**d**–**f**) and AFM-based Volta potential images (**g**–**i**) of three types of inclusions: lead-antimony-oxygen-rich inclusions (such as rosiaite) observed in the substrate of the Royal Summer Palace sample (**a**,**d**,**g**); Cu_2_O (cuprite)-rich inclusions in the substrate of the Kronborgs Castle material (**b**,**e**,**h**); copper-antimony-nickel-oxygen-rich inclusion in the substrate of the Mausoleum in Graz material (**c**,**f**,**i**).

In what follows, a mechanism is proposed to explain why the inclusion size may be of importance for the reduced uniformity of the patina and the increase in thickness ratio between the brochantite/antlerite sub-layer and the cuprite sub-layer, as indicated in Figure 5. The discussion starts by analyzing the electrochemical nobility of some larger inclusions found in the historic copper materials. From the elemental analysis of the inclusions analyzed (see Table 3), it is evident that at least three types of inclusions can be identified: lead-antimony-oxygen-rich inclusions (such as rosiaite) seen in, e.g., the Royal Summer Palace sample; cuprite-rich inclusions seen in the Kronborgs Castle substrate; and copper-antimony-nickel-oxygen-rich inclusions seen in the Mausoleum Graz substrate. These three types of inclusions were analyzed by AFM-based Scanning Kelvin Probe Force Microscopy to reveal the Volta potential of the inclusions relative to the surrounding copper matrix. The results are displayed in Figure 6.

The SEM-images, Figure 6a (lead-antimony-oxygen), Figure 6b (cuprite) and Figure 6c (copper-antimony-nickel-oxygen) show three representative inclusions. Their elemental composition has been analyzed by EDS. Each sample has then been transferred to an AFM-microscope where the same inclusions have been identified again (see Figure 6d–f). AFM-based Scanning Kelvin Probe Force Microscopy [22] was subsequently used to obtain Volta potential scans of each inclusion (see Figure 6g–i). As clearly seen, all inclusions show white areas in the Volta potential images indicating a relative nobility of those inclusion areas that is higher than the surrounding copper matrix [23].

**Figure 7:**
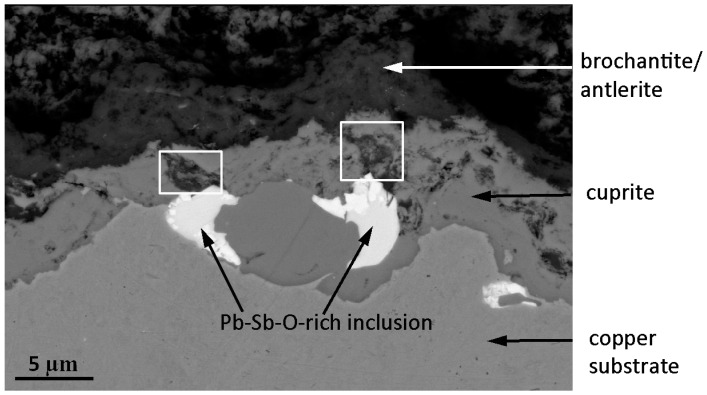
SEM cross-sectional image of the interfacial region between the copper substrate (lower part) and the patina (upper part) of the historic copper material from the Royal Summer Palace in Prague. White areas represent lead-antimony-oxygen-rich inclusions, light grey areas in the middle represent cuprite, and dark areas in the upper part brochantite/antlerite.

With a higher Volta potential it can be concluded that all three types of inclusions act as cathodes relative to the copper matrix. An immediate implication of this is that the inclusions during the atmospheric corrosion process trigger micro-galvanic corrosion effects in their immediate vicinity. This may cause not only accelerated corrosion kinetics but also a different sequence of corrosion product evolution than what would be expected without micro-galvanic effects [24], as illustrated in Figure 7. The figure displays a cross-section of the interfacial region between the copper substrate (light grey, lower part) and the patina (dark grey, upper part). As already shown in Figure 1 and Figure 2, the inclusions remain relatively intact during the oxidation of the copper metal surface forming a patina.

Figure 7 has captured the moment when two electrochemically more noble inclusions than the copper substrate (in this case two lead-antimony-oxygen-rich inclusions, white areas in the figure) have reached the copper matrix/patina interface. The grey areas in the figure represent cuprite, and the darker areas brochantite/antlerite. Near both lead-antimony-oxygen-rich inclusions the brochantite/antlerite layer is in direct contact with the inclusions (as marked by two squares in Figure 7), which has resulted in disrupture of the cuprite layer. A possible reason for this is the higher relative nobility of all inclusions relative to the copper matrix, which results in more oxidizing conditions and which therefore locally favors the formation of Cu^2+^-containing corrosion products (such as brochantite or antlerite) at the expense of Cu^+^-containing corrosion products (cuprite). It is well established that galvanic effects are favored by cathodic/anodic area ratios, and therefore not surprising that the largest inclusions result in the strongest micro-galvanic effects.

Therefore, the overall impact of the larger noble inclusions, such as those shown in Figure 7, is to disrupt the patina bi-layer structure, resulting in a more fragmentized patina and a brochantite/antlerite sub-layer, which, at least partially, grows at the expense of the cuprite sub-layer. This electrochemically-based influence of large inclusions on patina structure may not exclude other possible influences of the inclusions, e.g., the mechanical disrupture of the patina sub-layers. However, exploring this issue further requires complementary investigations.

**Figure 8:**
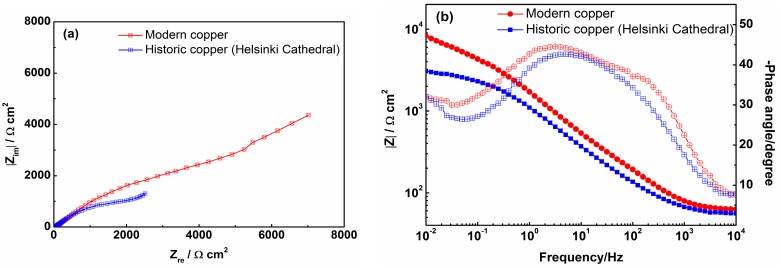
EIS spectra of Nyquist plot (**a**), impedance modulus (filled legend, (**b**)) and phase angle (unfilled legend, (**b**)) for the polished modern (DHP-Cu) and historic (Helsinki Cathedral) copper material during exposure in 0.1 M oxygen-free Na_2_SO_4_.

Considering the difference in microstructure between modern high-purity copper (e.g., DHP-Cu) and historic copper materials, and also the fact that the protective properties of the patina layer mainly have been attributed to the inner cuprite layer [21], the results suggests that high-purity copper has a superior corrosion protective ability over historic ones. A demonstration of this was performed by exposing DHP-Cu, which was diamond polished to 0.25 μm, and one of the historic samples (Helsinki Cathedral), which was also diamond polished to 0.25 μm after removal of the old patina layer, to an oxygen-free 0.1 M Na_2_SO_4_-solution. The results, as obtained with EIS, are displayed in Figure 8a (Nyquist plot) and Figure 8b (impedance and phase angle). The spectra all reveal two time constants, and an impedance modulus that is slightly higher for the modern copper material compared to the historic one throughout the whole frequency region. The results suggest a higher protective ability of the corrosion products formed on modern copper under current exposure conditions. However, considering the long lifetime of historical copper roofs, even “lower quality” copper exhibits an outstanding resistance to outdoor corrosion.

## 5. Conclusions

The historic naturally patinated sheet copper investigated herein shows an inhomogeneous patina with an outer layer of brochantite (or brochantite/antlerite) that is much thicker than the inner layer of cuprite. In modern sheet copper of higher purity (>99.9 wt %), on the other hand, the patina is more homogenous with comparable thicknesses of the two sub-layers.

Oxidic inclusions in the size range of up to 40 µm were observed. Three types of inclusions were identified: cuprite (Cu_2_O)-rich; rosiaite (PbSb_2_O_6_)-rich with a smaller fraction of cuprite; and oxides of a mixture of mainly copper, antimony and nickel.

The thickness ratio between the brochantite/cuprite sub-layers varied from 1.1 to 10.6 between the investigated materials of different age and exposure conditions. This thickness ratio seems to be directly related to the maximum size of the inclusions found in each historic material. The observation can be attributed to the higher relative nobility of all inclusions found, which creates micro-galvanic effects when the inclusions reach the substrate/patina interfacial region.

## Figures and Tables

**Table 1:** Exposure site, location (city, country) and approximate exposure period of investigated copper surfaces.

Exposure Site	City (Country)	Exposure Period (Years)
Royal Summer Palace (Belvedere)	Prague (Czech Republic)	~425
Mausoleum	Graz (Austria)	~390
Drottningholm Castle	Stockholm (Sweden)	~300
Basilica	Maria Dreieichen (Austria)	~160
Helsinki Cathedral	Helsinki (Finland)	~150
Kronborgs Castle	Elsinore (Denmark)	~120
Otto Wagner Church	Vienna (Austria)	~110
Church in Old Town	Stockholm (Sweden)	~100

**Table 2:** Density, size range in different directions, and elemental composition of selected inclusions in substrates of all investigated historic copper materials. bdl: below detection limit, around 0.5 atomic % or lower.

Exposure Site	Number of Inclusions/300 × 300 μm^2^	Size Range/μm	Elemental Composition/Atomic %						
			O	Cu	Pb	As	Sb	Ni	Sn
Royal Summer Palace, Prague	48 ± 9	2.1–38.2	66.8	4.0	10.7	bdl	18.5	bdl	bdl
Mausoleum, Graz	39 ± 11	2.5–14.8	52.9	26.4	bdl	bdl	7.3	13.4	bdl
Drottningholm Castle, Stockholm	73 ± 12	2.7–8.9	63.5	3.0	25.2	5.1	3.2	bdn	bdn
Basilica, Maria Dreieichen	62 ± 20	0.3–3.1	46.8	26.1	23.1	4.0	bdl	bdl	bdl
Helsinki Cathedral	18 ± 3	6.2–25.4	59.6	17.1	14.2	3.9	5.2	bdl	bdl
Kronborgs Castle, Elsinore	39 ± 11	1.5–4.8	34.8	65.2	bdl	bdl	bdl	bdl	bdl
Otto Wagner Church, Vienna	57 ± 18	0.5–8.6	67.7	1.5	14.6	0.7	6.3	bdl	9.2
Church in Old Town, Stockholm	35 ± 5	3.1–15.5	65.7	1.4	23.9	7.8	1.2	bdl	bdl

**Table 3:** Approximate average thicknesses with standard deviation (μm) of the two sub-layers and the corresponding data for the ratio of brochantite/antlerite sub-layer and cuprite sub-layer. Data in brackets show corresponding minimum and minimum thickness values (μm). The data were obtained through measurements of five different cross-sections of the patina using SEM images (≥90 μm × 65 μm) for each material.

Exposure Site	Thickness of Cuprite (t_1_)	Thickness of Brochantite/Antlerite (t_2_)	t_2_/t_1_
Royal Summer Palace, Prague	4.2 ± 3.3 (1.2, 8.2)	42.5 ± 11.8 (30.7, 52.5)	10.6 ± 6.1
Mausoleum, Graz	9.1 ± 4.4 (3.3, 13.7)	13.3 ± 14.5 (7.8, 33.8)	1.5 ± 1.4
Drottningholm Castle, Stockholm	9.5 ± 6.1 (3.4, 17.4)	23.4 ± 11.3 (11.7, 30.2)	2.5 ± 1.5
Basilica, Maria Dreieichen	9.9 ± 6.9 (3.1, 15.5)	11.2 ± 5.2 (6.1, 17.5)	1.2 ± 0.9
Helsinki Cathedral	1.1 ± 0.7 (0.8, 1.8)	8.7 ± 2.1 (6.7, 9.1)	8.1 ± 3.2
Kronborgs Castle, Elsinore	4.1 ± 3.9 (1.0, 8.1)	15.4 ± 6.2 (9.5, 22.9)	3.7 ± 2.6
Otto Wagner Church, Vienna	9.7 ± 4.8 (4.4, 16.1)	10.6 ± 6.8 (5.5, 17.9)	1.1 ± 0.9
Church in Old Town, Stockholm	10.3 ± 6.6 (3.6, 13.2)	17.4 ± 5.9 (14.4, 24.3)	1.6 ± 1.5
